# Bioactive Molecules Derived from Plants in Managing Dengue Vector *Aedes aegypti* (Linn.)

**DOI:** 10.3390/molecules28052386

**Published:** 2023-03-05

**Authors:** Sridhar Shanmuga Priya, Prabhakaran Vasantha-Srinivasan, Ammar B. Altemimi, Ramji Keerthana, Narayanaswamy Radhakrishnan, Sengottayan Senthil-Nathan, Kandasamy Kalaivani, Nainarpandian Chandrasekar, Sengodan Karthi, Raja Ganesan, Zina T. Alkanan, Tarun Pal, Om Prakash Verma, Jarosław Proćków

**Affiliations:** 1 Department of Biotechnology, St. Peter’s Institute of Higher Education Research, Chennai 600077, India; 2 Department of Bioinformatics, Saveetha School of Engineering, Saveetha Institute of Medical 17 and Technical Sciences (SIMATS), Chennai 602105, India; 3 Department of Food Science, College of Agriculture, University of Basrah, Basrah 61004, Iraq; 4 College of Medicine, University of Warith Al-Anbiyaa, Karbala 56001, Iraq; 5 Department of Biotechnology, RV College of Engineering, Bangalore 560059, India; 6 Department of Biochemistry, Saveetha Medical College and Hospital, Saveetha Institute of Medical and Technical Sciences (SIMATS), Chennai 602105, India; 7 Division of Bio-Pesticides and Environmental Toxicology, Sri Paramakalyani Centre for 14 Excellence in Environmental Sciences, Manonmaniam Sundaranar University, Alwarkurichi, 15, Tirunelveli 627412, India; 8 Post Graduate and Research Centre, Department of Zoology, Sri Parasakthi College for Women, Courtrallam 627802, India; 9 Centre for Geo–Technology, Manonmaniam Sundaranar University, Tirunelveli 627012, India; 10 Department of Entomology, College of Agriculture, Food and Environment, University of Kentucky, Lexington, KY 40503, USA; 11 Institute for Liver and Digestive Diseases, College of Medicine, Hallym University, Chuncheon 24252, Republic of Korea; 12 Department of Biotechnology, Vignan’s Foundation for Science, Technology and Research, Vadlamudi, Guntur 522213, India; 13 Department of Molecular and Cellular Engineering, Jacob Institute of Biotechnology and Bioengineering, Sam Higginbottom University of Agriculture, Technology and Sciences, Prayagraj 211007, India; 14 Department of Plant Biology, Institute of Environmental Biology, Wrocław University of Environmental and Life Sciences, Kożuchowska 5b, 51-631 Wrocław, Poland

**Keywords:** *Aedes aegypti*, plant crude extracts, metabolites, larvicidal, pupicidal, adulticidal, ovicidal, oviposition deterrent, non-target toxicity

## Abstract

Mosquitoes are the potential vectors of several viral diseases such as filariasis, malaria, dengue, yellow fever, Zika fever and encephalitis in humans as well as other species. Dengue, the most common mosquito-borne disease in humans caused by the dengue virus is transmitted by the vector *Ae. aegypti*. Fever, chills, nausea and neurological disorders are the frequent symptoms of Zika and dengue. Thanks to various anthropogenic activities such as deforestation, industrialized farming and poor drainage facilities there has been a significant rise in mosquitoes and vector-borne diseases. Control measures such as the destruction of mosquito breeding places, a reduction in global warming, as well as the use of natural and chemical repellents, mainly DEET, picaridin, temephos and IR-3535 have proven to be effective in many instances. Although potent, these chemicals cause swelling, rashes, and eye irritation in adults and children, and are also toxic to the skin and nervous system. Due to their shorter protection period and harmful nature towards non-target organisms, the use of chemical repellents is greatly reduced, and more research and development is taking place in the field of plant-derived repellents, which are found to be selective, biodegradable and harmless to non-target species. Many tribal and rural communities across the world have been using plant-based extracts since ancient times for various traditional and medical purposes, and to ward off mosquitoes and various other insects. In this regard, new species of plants are being identified through ethnobotanical surveys and tested for their repellency against *Ae. aegypti*. This review aims to provide insight into many such plant extracts, essential oils and their metabolites, which have been tested for their mosquitocidal activity against different life cycle forms of *Ae. Aegypti*, as well as for their efficacy in controlling mosquitoes.

## 1. Introduction

Mosquitoes are one of the major vectors that carry harmful viruses that spread fatal and deadly diseases all over the world [1]. Compared to all the other arthropods, mosquitoes are responsible for spreading the highest number of mortal diseases like dengue, chikungunya, elephantiasis, malaria, etc. [2]. They are responsible for spreading vector-borne diseases to more than 1 billion people every year. Over 1 million people die every year due to these diseases [3]. The survival and transmission of viral pathogens is entirely dependent on the vectors carrying the pathogens. Hence, mosquitoes play a major role in the pathology of diseases [4]. Mosquitoes are considered to be the greatest enemy of the human race as they greatly affect public health, spread deadly diseases, and are major competitors in aquatic and terrestrial food chains [5]. There are no significantly specific treatments for these arboviral diseases and finding eco-friendly ways of eradicating the vector is a more efficient way to control the disease [4]. With the Zika fever outbreak spreading across Latin America, many travelers and people in endemic areas are left wondering how best to protect themselves from mosquitoes. The tropical yellow fever mosquito, *Ae. aegypti*, is the primary vector for transmitting dengue. It is a day-feeding mosquito that has major epidemiological significance, as it is responsible for spreading several other arboviral diseases like chikungunya and the Zika virus. Other mosquitoes, such as *Aedes albopictus*, also called the Asian tiger mosquito, can also act as carriers [6,7].

Of these arboviral diseases, dengue is one of the fastest re-emerging diseases that greatly affects the economy and health of many countries [8]. Dengue is an acute viral disease in humans caused by a single-stranded RNA virus that belongs to the Flaviviridae family. Although the symptoms are clinically in apparent most of the time, they may lead to severe manifestations like DSS or DHF [9,10]. A cartographic study states that there are about 390 million dengue infections per year, of which 96 million cases show significant clinical symptoms [9]. Dengue does not have a particular treatment, but there is a chimeric tetravalent vaccine that is made of attenuated serotypes of dengue viral strains [11]. Many products claim to deter mosquitoes, but not many have been scientifically proven effective. Usually, the mosquito larvae are targeted, and their growth is hindered by using organochlorine, organophosphates, or growth regulators. However, these methods have a negative impact on the environment [7]. Recent research shows that silver nanoparticles obtained from plants can effectively act against dengue serotypes DEN-2 and *Ae. aegypti* [12]. In order to control the vector population, STI combined with auto-dissemination has been highly efficient and does not have any detrimental effect on the environment [13]. Most commercially available mosquito repellents contain one or several active ingredients, including N,N-diethyl-meta-toluamide, commonly known as DEET, IR3535, or picaridin. A recent study tested commercially available mosquito repellents against both *Ae. aegypti* and *Ae. albopictus*. Those sprays containing DEET were the most effective, repelling both species with >70% efficacy for at least four hours [14]. Methods like covering open water containers and introducing fish that feed on mosquito larvae have been found to be efficient in reducing the population of mosquito vectors [15]. Nevertheless, the use of fish that feed on mosquito larvae affects the ecosystem and disrupts the food chain by affecting the native aquatic fauna [16]. The use of insecticides that kill the larval and pupal stages of mosquitoes is more efficient, but over a period of time, the insects develop resistance to the insecticide by increasing their metabolism and enzyme activity [17,18,19]. In addition, the use of insecticides has a negative impact on the environment and non-target organisms [20]. Later, biorational pesticides were developed based on dose response studies with target and non-target species, but this method also later proved to affect the non-target organisms [21]. 

While DEET is an active ingredient in many repellents, several reports linking DEET use to negative health effects have resulted in health changes, external pubic disorders and chemophobia [22]. However, research shows that with an estimated 200 million applications of DEET occurring every year, there have been only 14 reported incidents of adverse effects, and most were the result of overuse [23]. Concerns about the negative effects of DEET have contributed to the popularity of repellents composed of plant-based compounds. 

The traditional mosquito control strategy focused on killing the mosquitoes using different types of insecticides. An eco-friendly approach, through declining or eliminating the breeding sites of mosquito, has often been used along with microbiological larvicides, ovicides and pupicides [8]. Traditionally, insect repellents work by providing a vapor barrier deterring mosquitoes from meeting the skin surface. Over the past thousand years, insect repellents have been utilized to stop arthropods from biting. Several species of primates were experimentally daubing their pelage by rubbing it with plants, including *Piper marginatum*, Citrus spp. and *Clematis dioica*, native to south Asian and American countries [7]. Naturally derived repellents from plants belong to the Asteraceae, Cupressaceae, Lamiaceae, Lauraceae, Labiatae, Myrtaceae, Meliaceae, Poaceae, Piperaceae, Umbelliferae, Rutaceae and Zingiberaceae families [8]. They have been evaluated for repellency against different mosquito vectors, but few compounds have been found to be commercially available. The public deliberates herbal-based repellents as harmless and the right alternative to chemical pesticides; most of them are manufactured and distributed through local herbal harvesters and suppliers and have been seen on the market for a minimal period. Despite having many botanical based pieces of research, almost all registered plant-based commercial repellents and their active compounds deliver a limited time of protection and require recurrent reapplication [5]. Increased curiosity in plant-based arthropod repellents was generated after the United States Environmental Protection Agency (USEPA) added a rule to the Federal Insecticide, Fungicide and Rodenticide Act (FIFRA) in 1986. Formulations containing essential oils are frequently found in the active ingredients of mosquito repellents marketed as “organic” or “natural”. Despite their newfound popularity, these alternatives are largely ineffective when compared to DEET. However, research by [14] found that DEET-free formulations did not perform as well as those containing DEET, products containing only essential oils from lemongrass, citronella, soybean, rosemary and cinnamon provided brief repellency immediately upon application. However, within four hours after application, the sprays were no longer repellent or had substantially reduced repellency. Plant-based products can provide effective protection, but their effect is usually temporary compared to solutions containing synthetic chemicals, which tend to breakdown less easily. The different herbal extraction methods of essential oil for repellents has been displayed (Supplementary Figure S1).

Humans have been using plants for medicinal purposes since the birth of civilization. The oldest documentation of this dates back to around 5000 years ago in Nagpur, India. A piece of Sumerian clay has been found, upon which 12 different recipes for the preparation of drugs are mentioned, which refer to over 250 plants. Among them are poppy, henbane and mandrake [24]. It is likely that medicinal plants have been used for a great deal longer, and there are documented cases of apes and monkeys using different plants for medicinal purposes. In Costa Rica, plants of the pepper genus are seen being rubbed on the fur to repel parasites. This is something that is also being used by the human population, especially *Piper auritum* [25]. Following the discovery of the Sumerian clay piece in Nagpur, many other recordings have followed up to the present day. As a result, medicinal plants have had and continue to have a significant impact on health care, paralleling the synthetic pharmaceutical industry around the world. The Lao PDR is very interesting with regard to ethnobotany. Laos is a multi-ethnic country with a long tradition of using medicinal plants that is very much alive even today [26]. The country is relatively unharmed by deforestation and has therefore conserved a large portion of its biodiversity. In 2010, Dr. Hugo de Boer and his colleagues conducted interviews in 66 villages in Laos and established the use of 92 different plant species for repelling a variety of hematophagous parasites [27]. 

Herbal-based extracts or essential oils have been involved in a significant part of managing vector-borne diseases with the discovery of unique phytocompounds from individual bio-active extracts or essential oils. Among the different natural extracts or bio-active compounds, flavonoids have lately been given substantial attention by herbal researchers due to their favorable chemo-protective abilities in different treatments including for cardiovascular diseases, neurodegenerative disorders, inflammatory disorders, diabetes, malaria, dengue and other deadly microbial infections [27]. Since then, we have conducted further interviews in northwestern Laos, acknowledging further species. Together with further extensive study of the existing literature, a selection of plant candidates for repelling *Ae. aegypti* emerged.

## 2. Virus Vector

It has long been known that parasitic arthropods may act as vectors for transmitting certain viruses. The *Ae. aegypti* mosquito is a recognised vector for DENV, CHIKV and YFV [28]. These diseases account for 454–900 million cases annually worldwide [3]. Yellow fever is not present in the Lao People’s Democratic Republic (Laos) and will therefore not be in focus within this report. The *Ae. aegypti* is a small, day-active, black mosquito with white stripes on its joints. As with all mosquitoes, it is only the female mosquitoes that show hematophagy behaviour. 

### 2.1. Life Cycle of Aedes aegypti

Adult *Aedes* mosquitoes are different from other types of mosquitoes due to the fact that they have narrow black bodies that absorb all radiation falling on them. Unique alternating patterns of light and dark scales are predominantly seen on their abdomen, thorax and legs. The females possess tapering abdomen and maxillary palps (shorter than the proboscis), which distinguish them from their male counterparts [29]. 

**Figure 1:**
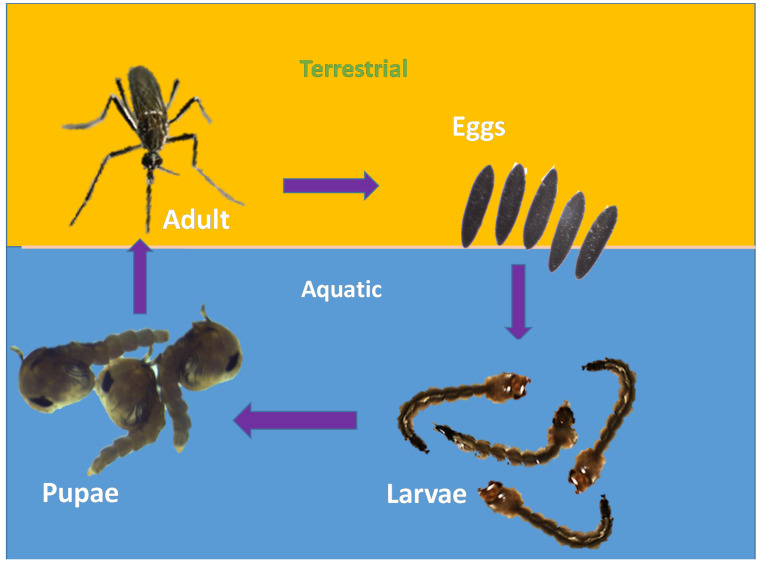
Life Cycle of *Aedes aegypti*.

*Ae. aegypti* have four life stages namely (Figure 1):

- Eggs: About 100 black-coloured eggs are laid by adult female mosquitoes on a wet/moist surface very near the waterline, especially in places such as marshes, plant axils, tree holes and even water containers [29]. Man-made objects such as clay pots, bowls, cups, fountains, barrels, vases and tires are excellent sites for egg laying [30]. The eggs are very hardy and become glued to the wet surface. Due to their ability to endure long periods of drying, they can survive extreme cold and other adverse climatic conditions;
- Larvae: The emergence of larvae from the eggs takes place only after they get fully immersed in water. The process might take days to weeks and some of the eggs require multiple soakings before they hatch. The larvae are aquatic. They hang upside down at an angle from the water surface [29]. They feed on the microorganisms found in water [30]. Siphon is their short respiratory structure, through which they take up oxygen from the air above the water [30]. After undergoing the process of moulting thrice, a larva becomes a pupa.
- Pupa: It takes a larva five days to become a pupa. The pupa continues to develop until the body of the adult mosquito emerges from the pupal skin and exits the water [30];
- Adult: After around 2–3 days, the adult emerges from the pupa [30]. Within two days of emerging, adult mosquitoes mate. Male mosquitoes feed on nectar found in flowers, whereas female mosquitoes consume their blood meal. Though they feed during daytime, their activity peaks at dawn and dusk [29]. After feeding, the mosquitoes look for water surfaces to lay their eggs [30]. They usually prefer to live in close association with humans [29], especially inside homes and buildings where the windows and doors are kept open [30].

### 2.2. Role in Transmission of Diseases

A number of diseases are transmitted by *Ae. aegypti*. The major ones are:

#### 2.2.1. Dengue Fever

It is estimated that there are about 100,000 cases of dengue fever in Laos alone, annually. The total costs for these episodes every year is believed to be more than USD 5 million [31]. In a low-income country, such as Laos, this amount to a substantial sum of money, which could have been utilized in a developmental manner. DENV comes in four serotypes: 1, 2, 3 and 4, which are very closely related, thus serological tests suggest cross-reactivity. Yet, cross-protective immunity does not seem to occur [32]. After a mosquito carrying the virus bites a person, the subject undergoes a 3–14 day incubation period. Directly following is the acute onset of fever accompanied by a variety of common influenza symptoms, depending on the serotype. These may include headache, retro-orbital pain, joint pains, weakness, nausea and vomiting. The febrile period may last 2–10 days, while the virus circulates in the subject’s blood. This is usually followed by a brief period of relative recovery before the recurrence of symptoms, such as nosebleeds, circulatory failure and a distinctive rash that begins peripherally and spreads to the thorax and back [32,33].

#### 2.2.2. Chikungunya

As the name implies Chikungunya fever is an acute febrile illness, caused by the virus CHIKV. The emergence of CHIKV seems to be cyclical [34,35]. It re-emerged in Kenya in 2004 and has subsequently, spread to novel areas such as Europe, maybe due to an increasingly warmer climate. It has since caused millions of disease cases throughout the globe [34]. Symptoms include high fever and arthralgia or severe joint pain that occurs in almost all patients. 

Most infections are resolved within a few weeks but there are reports of cases lasting for many months with recurring episodes of symptoms [36]. The risk of death is about 0.2%. Metz et al. from TI Pharma claimed in 2013 that they had developed a working vaccine against the virus. Refining and distributing such a product is costly and takes time, thus, Chikungunya fever may still be regarded as a health issue that may be eluded by botanical repellents [8]. 

#### 2.2.3. Zika

India is at risk of Zika virus transmission due to the high prevalence of its vector, *Ae. aegypti*. Rajasthan, a state in the northwest region of India, also has a high prevalence of the Aedes mosquito. An explosive Zika epidemic was reported in Brazil in 2015. Though the WHO declared that ZVD ceased to be a Public Health Emergency of International Concern after November 2016, GOI continued to be on high alert due to an abundance of the vector *Ae. aegypti* and high international travel from endemic countries. The ICMR initiated ZIKV surveillance through its network of VRDL, with the NIV as the apex laboratory from 2016. As evident from the present cases, Zika may not be a recent introduction in India. In 1954, the NIV, Pune (then the Virus Research Centre), had tested samples from the Bharuch district, which showed ZIKV antibody detection in 16.8 percent of the samples (Emergencies Preparedness, Response. Zika Virus Infection India) [37].

## 3. Protection

**Figure 2:**
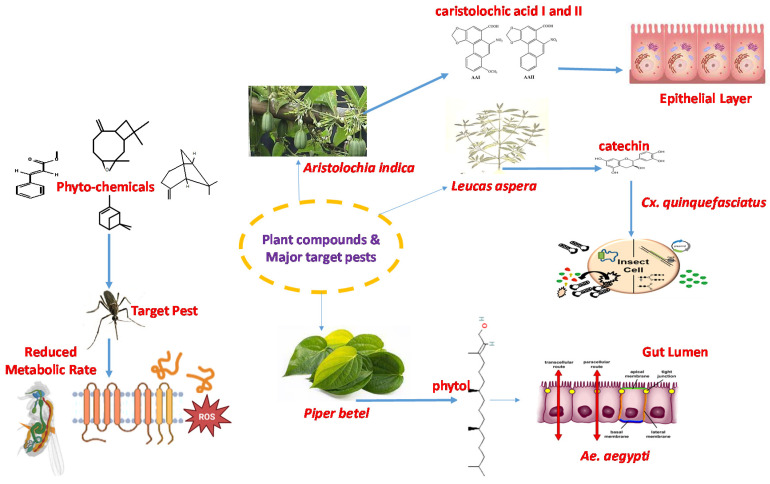
Mode of action of plant metabolites on mosquito gut cells.

Currently, there are no effective vaccines against DENV, nor are there any successful treatments when infected except for the relief of symptoms [3]. Preventive measures are the most effective way to combat DENV and CHIKV today. Using repellents that are effective against *Ae. aegypti* is a successful approach to avoiding bites, which would otherwise lead to infection risk. According to the World Bank, in 2008, 27.6% of the population in Laos lived below the poverty line, meaning one must sustain life on USD 1.25 or less per day. This generally means that synthetic insect repellents are not prioritised. Synthetic repellents in Laos today are almost exclusively imported from Thailand and are based on DEET. A recent study by Corbel and colleagues showed the inhibition of cholinesterase and possibly another neurotoxicity in both insect and mammalian nervous systems by DEET. This further supports the idea of utilising an innovative, safe and plant-based solution. A schematic view of the mode of action of a plant metabolite in the mosquito gut is displayed (Figure 2). 

## 4. Chemical Repellents Currently in Use against *Aedes aegypti*

Ranging from spraying aerosols, applying lotions, using coils, curtains, and clothes treated with active insecticidal compounds, mass fogging, and use in breeding places, chemical insecticides play a major role in the control of mosquitoes and have been very effective [38]. These insecticides potentially target all the life cycle forms of *Ae. aegypti* and are divided into larvicidal, adulticidal and pupicidal categories, based on their activity against the larval, adult and pupal stages of *Ae. aegypti.*

The four classes of insecticides recommended by the WHO for indoor residual spraying are pyrethroids (permethrin, sumithrin and deltamethrin), carbamates (carbosulfan and carbaryl), organophosphates (malathion, naled and temephos) and organochlorines (DDT, DDD, dicofol, aldrin, dieldrin, and chlorobenzoate). Owing to their high efficacy, low mammalian toxicity and short residual action, pyrethroids are the only insecticides approved for use on long-duration insecticidal nets against mosquito vectors [39]. Some of the most common insect repellents currently in use include DEET, IR-3535 and picaridin. These repellents can be used on clothes, as well as on the skin.

## 5. Necessity of Natural Mosquito Repellents 

Repeated use of the active ingredients in the repellents causes the mosquitos to develop resistance against them. Such resistance development has been reported in several countries, such as Colombia, Brazil, Grand Cayman, Thailand, India, Malaysia, Mexico and China [38]. The two main reasons for resistance development are (i) knockdown of the gene that encodes for the target binding site of the insecticides (*kdr* mutation sodium channel resistance) and (ii) upregulation of the mosquito detoxifying enzymes [39]. Most of these repellents cause allergic reactions such as swelling, rashes and eye irritation in adults and children, and offer protection only for 2 to 4 h [40]. Though DEET has good efficacy and offers better protection, it is found to be toxic to the skin and nervous system, as it inhibits ion channels and human acetylcholinesterase and is involved in the modulation of G-protein-coupled receptors [41]. Apart from causing pollution in the environment, these repellents bioaccumulate and are sometimes toxic to non-target species.

Plant-based insecticides (biopesticides) are found to be selective, biodegradable, sustainable and cause little or no harm to non-target organisms [42]. Due to its agro-climatic conditions, India has the greatest resource of medicinal plants and is therefore considered to be the botanical backyard of the world [43]. Plant-based repellents have been exploited by men for thousands of years. The hanging of leaves of certain plants in front of the house, using dried, burnt leaves to ward off mosquitoes, and applying oils or formulations to the skin and clothes are still being practised in many countries, and have also been recorded in the writings of Indian, Greek and Roman scholars [44]. Many rural and tribal communities in India and several other tropical countries rely on plant-based repellents as the only means of protection against mosquito bites owing to their poverty [44].

## 6. Identifying and Screening New Plants for Repellency 

On the basis of knowledge of ethnobotany, new plant-based repellents could be discovered. Ethnobotanical surveys involve the targeted search for medicinal plants by conducting structured interviews with informants well-versed in folklore and traditional medicine, in combination with the collection of plant voucher specimens [44]. Indigenous ethnic groups are questioned on topics related to plant sources, usage and abundance. This method is easier and more direct when compared to a general screening of all plants in a particular area [44]. Bioprospecting is another method wherein there is a systematic screening of plants based on their mode of action. This process is labour intensive and expensive. PMD, an effective insecticide, was discovered in the 1960s through the process of mass screening of plants [44]. However, in recent times, with the support of bioinformatics tools, such as NCBI and many other databases, the search for new repellent plants has proven to be effortless, cost effective and less time consuming. 

## 7. Plants Tested for Larvicidal Activity against *Aedes aegypti*

The crude extracts of leaves, flowers, fruits, rhizomes and endosperms of plants exhibited significant larvicidal activity against *Ae. aegypti*, which was measured by lethal concentration (LC) and larval mortality. A few examples of such crude extracts and their efficacies are given (Table 1). 

The ethanolic seed extract of *Annona mucosa* (Annonaceae) produced an LC_50_ value of 2.6 mg/mL when exposed to *Ae. aegypti* larvae for 24 h [47]. At a concentration of 500 mg/mL, the dichloromethane extract of the leaves of *Ateleia glazioviana* (Fabaceae) produced high larvicidal activity [59]. The LC_50_ values of the aqueous extract of another plant of the family Annonaceae, *Annona glabra*, when exposed to *Ae. aegypti* larvae for 24 and 48 h were 2.43 mg/L and 1.17 mg/L, respectively [50]. The petroleum ether extracts of leaves of three plants, namely *Hyptis suaveolens* (Lamiaceae), *Lantana camara* (Verbenaceae) and *Tecoma stans* (Bignoniaceae), had LC_50_ values of 64.49, 74.93, and 84.09, respectively, and were found to be toxic against *Ae. aegypti* larvae [60]. The blend of these four extracts had an LC_50_ of 7.19 mg/L and a predator safety factor (for *Gambusia affinis*) of 12.55. Hence, these extracts and their blend were safe for non-targets [60]. 

The methanolic extracts of petals, leaves and roots of *Ipomoea cairica* (Convolvulaceae) showed potential larval mortalities, with LC_50_ values of 12.7, 13.6, and 31.9 mg/L, respectively, against *Ae. aegypti* [61]. The methanolic extracts of *Persea americana* (avocado) (Lauraceae) unripe fruit peel [62], *Nerium oleander* (Apocynaceae) leaves [60], *Rubia cordifolia* (Rubiaceae) roots [63], *Argemone mexicana* (Papaveraceae) seed [64] and *Sida acuta* (Malvaceae) leaves [65], displayed good larval toxicity with LC_50_ values (after 24 and 48 h of exposure) of 7.12ppm, 84.09 mg/L,102.23 mg/L, 80 mg/mL and 42.08 mg/L respectively. The plant extracts of *Argemone mexicana*, when screened, were found to be abundant in metabolites, such as alkaloids, flavonoids, coumarins, saponins, tannins, cardiotonics, glycosides, sterols and terpenes [64]. 

Significant larval mortalities were observed in the methanolic extracts of *Anacardium occidentale* (Anacardiaceae), *Dianella longifolia* (Liliaceae), *Litsea leefeana* (Lauraceae), *Trenica grandifolia* (Ulmaceae), *Canthium gueinzii* (Rubiaceae), *Kigelia pinnata* (Bignoniaceae), *Rumex obtusifolius* (Polygonaceae) and *Ruta chalepensis* (Rutaceae), at a concentration of 400 ppm when exposed to *Ae. aegypti* larvae for 24 h [66].

An increase in the concentration of ethanolic extract from the leaves of *Momordica charantia* (Cucurbitaceae) enhanced the larval mortality of *Ae. aegypti* [51]. In terms of the mortality rate, different extracts of the leaves of *Pavetta tomentosa* and *Tarenna asiatica*, both belonging to the family Rubiaceae, produced dissimilar effects, with the highest toxicity exhibited by the ethyl acetate extract and the lowest toxicity exhibited by the chloroform extract against *Ae. aegypti* [52]. Similarly, the crude extracts of *Ambrosia arborescens* (Asteraceae) leaves [53], *Scilla peruvina* (Asparagaceae) roots [63], *Anamirta cocculus* (Menispermaceae) endosperms [67], *Acorus calamus* (Acoraceae) fresh rhizome [68] and *Limonia acidissima* (Rutaceae) leaves [57], showed remarkable larval toxicity, with LC_50_ values of 1844.61 ppm, 114.13 mg/L, 56.73 mg/L, 57.32 mg/L and 4.11 ppm, respectively, after 24 h exposure to the larvae. The acetone, petroleum ether and ethanol extracts of *Tribulus terrestris* (Zygophyllaceae) leaves after just a 4 h exposure to the larvae produced LC_50_ values of 173.2, 64.6 and 376.4 ppm, respectively, and thus, all three extracts proved to be effective [69].

The ethanolic extracts and metabolites of *Annona mucosa* (Annonaceae) were found to be non-toxic when tested against a non-target organism, such as zebrafish, and hence, could be further developed as potential mosquito repellents with a good safety factor [47]. Not only the crude extracts but also some of the essential oils extracted from the leaves and other parts of plants, such as those from *Croton nepetaefolius* (Euphorbiaceae) and *Syzygium aromaticum* (Myrtaceae), were larvicidal in nature, as they produced 50% larval mortalities at concentrations of 32.7 and 81.7 ppm, respectively [51]. The essential oil derived from *Anethum graveolens* (Zingiberaceae) fruit, at 10% concentration, offered 100% larval mortality with a LC_50_ of −0.3% after a 72 h exposure period [70]. 

**Figure 3:**
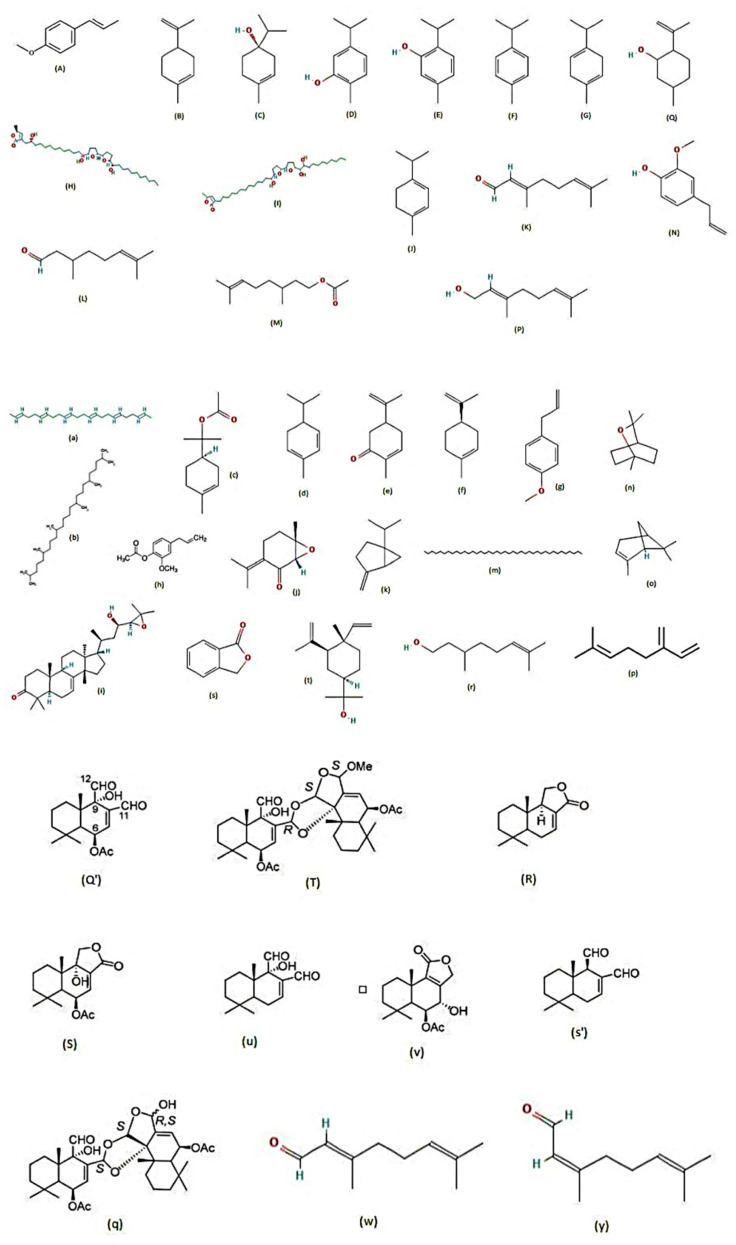
Chemical structures of major metabolites detected in larvicidal plants [72,73,74,75,76,77,78]. (**A**) trans-anethole, (**B**) limonene, (**C**) terpinen-4-ol, (**D**) carvacrol, (**E**) thymol, (**F**) p-cymene, (**G**) γ-terpinene, (**H**) rolliniastatin-1, (**I**) rollinicin, (**J**) α-terpinene, (**K**) citral, (**L**) citronellal, (**M**) citronellyl acetate, (**N**) eugenol, (**P**) geraniol, (**Q**) isopulegol, (**Q’**) CDIAL, (**R**) CML, (**S**) CMOS, (**T**) CFRAG, (**a**) 2,6,10,14,18,22-tetracosane hexane, (**b**) 2,6,10,15,19,23-hexamethyltetracosane, (**c**) 3-cyclohexene-1-methanol,.alpha., .alpha.4-trimethyl, (**d**) α-phellandrene, (**e**) carvone, (**f**) d-limonene, (**g**) estragole, (**h**) eugenyl acetate, (**i**) niloticin, (**j**) rotundifolone, (**k**) sabinene, (**m**) tetracontane, (**n**) 1,8-cineole, (**o**) α-pinene, (**p**) β-myrcene, (**q**) CPCD, (**r**) citronellol, (**s**) phthalide, (**s’**) POLYG, (**t**) elemol, (**u**) WARB, (**v**) UGAN, (**w**) geranial, (**y**) neral.

Three plants from the family Canellaceae, namely *Cinnamosma fragrans*, *Cinnamosma madagascariensis* and *Warburgia ugandensis*, were examined by [71] for their larvicidal activity against *Ae. aegypti*. Their bark, roots and leaves were used for extraction, and many metabolites were detected such as CDIAL, UGAN, CPCD, and POLYG from *C. fragrans*; CMOS, Cinnafragrin A, and CML from *C. madagascariensis*; and WARB (a sesquiterpene dialdehyde) from *W. ugandensis*. The larvicidal activity was proportional to the amount of CDIAL and POLYG present, and in the case of *C. fragrans*, 60% CDIAL was found in the bark, 30% CDIAL in the root extracts and no detectable CDIAL in the leaf extract. In *C. madagascariensis*, the bark and root extracts showed almost 75% larvicidal activities, while the leaf extract had approximately 0.9% efficacy. Of all the metabolites identified, WARB and CDIAL exhibited the strongest toxicity against *Ae. aegypti* larvae, with POLYG being moderately toxic and CML and CMOS being nominally toxic [71]. The major phytocompounds that display larvicidal activity are displayed (Figure 3). 

## 8. Plants Tested for Repellent Activity against *Aedes aegypti*

The methanolic extracts of *Sonnerita alba* (Lythraceae), *Avicennia marina* (Acanthaceae), *Avicennia alba* (Acanthaceae), *Rhizophora stylosa* (Rhizophoraceae) and *Rhizophora apiculata* (Rhizophoraceae) showed excellent repellence of 85.8%, 80.5%, 80.5%, 81.3% and 80.3% for a period of 2 h, 3 h, 1 h, 1 h and 2 h, respectively, when exposed to *Ae. aegypti* for 6 h [79]. They are mangrove plants, which are rich in compounds such as alkaloids, flavonoids, saponins, steroids, quinones, phenols, triterpenoids and glycosides [79].

Lotions were prepared from the essential oils of *Citrus aurantifolia* (Rutaceae) leaves, *Citrus grandis* (Rutaceae) fruit peels and *Alpinia galanga* (Zingiberaceae) rhizomes, through microencapsulation [80]. At a concentration of 20%, all three lotions offered complete protection against *Ae. aegypti* for 2 h. At a concentration >90% the protection was extended to 4 h. The A. galanga-based formulation exhibited the highest protection of 98.91% for 4 h post-application [80].

The crude extracts of *Clausena anisata* (Rutaceae) leaves were examined for three types of repellency, namely: topical-based repellency, repellent-treated nets and space spraying assays [81]. The repellency over 3 h was found to be 83% for the acetone crude extract (with a concentration of 15%) and 54% at 7.5% concentration for the hexane extract. For mosquito bites, repellency was found to be 93% for the crude extract and 67% for the hexane fraction. Butanol and chloroform were found to be ineffective at any of the concentrations [81]. As with treated nets, acetone and hexane extracts of *Clausena anisata* had a repellency of 46.89% and 50.13%, respectively, after 3 h. Finally, as nebulisers, the EC50 of these extracts were 78.9 and 71.6 mg/mL, respectively, and they caused mosquito knockdown and, eventually, death [81]. The essential oils are extracted from the fruits of plants in the Rutaceae family, specifically *Citrus aurantifolia*, *Citrus aurantium*, *Citrus hystrix*, *Citrus maxima*, *Citrus medica*, *Citrus reticulata* and *Citrus macrocarpa*, which have been shown to repel *Ae. aegypti* [82]. The order of repellency of their essential oils (EOs) was as follows: *C. aurantifolia* > *C. microcarpa* > *C. maxima* > *C. reticulata* > *C. hystrix* > *C. aurantium* > *C. medica*. All the EOs had a longer protection time than the chemical repellent IR3535 (Johnson’s Baby Cream). *C. aurantifolia* had the highest repellency, with a CPT, biting rate and % protection of 65 min, 1.5%, and 98.5% protection, respectively [82]. 

At a concentration of 5 mg/cm2, the methanolic extract of the leaves of *Sida acuta* (Malvaceae) provided 100% protection for 180 min against *Ae. Aegypti*. The repellency was dependent on the concentration of the crude extract [65].

The protection time was found to be directly related to the concentration of the essential oil in the case of *Clausena dentata* (Rutaceae) [83]. At a concentration of 2.5%, the mean protection time was 180 min (the lowest), while at a concentration of 10%, the mean protection time had increased to 255 min. The essential oil of *C. dentata* was also found to be skin friendly, as its mean score for the skin potential test was zero [83]. 

**Figure 4:**
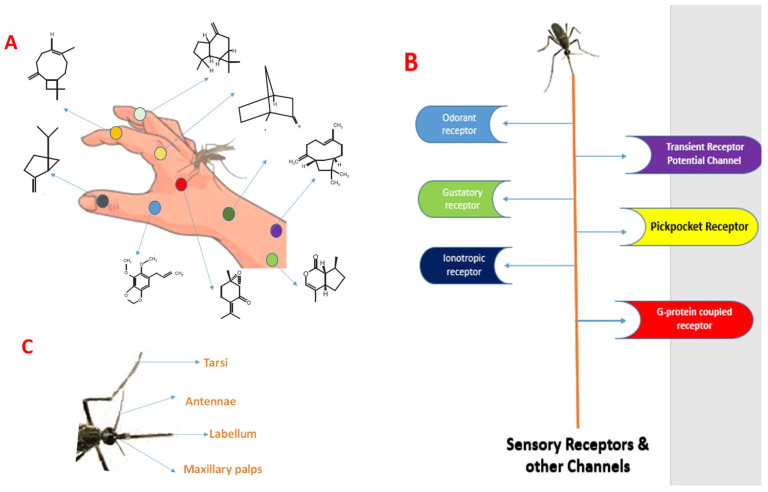
(**A**) Dengue mosquitoes use chemical cues to find a host and feed. Also, plant repellents with different phytocompounds with different modes of action. (**B**) Repellent’s response to specific molecular targets including sensory receptors (odorant receptor (OR), gustatory receptor (GR) and ionotropic receptor (IR)) distributed on various arthropod appendages. Future insect repellents may interact with other receptor families including the transient receptor potential channel (TRP), pickpocket receptor (PPK) and G-protein-coupled receptor (GPCR). (**C**) Sensory appendages of mosquito vector.

Generally, repellents may task at a distance (spatial repellents likely pointing the olfactory system) or contact (likely starting gustatory or other sensory systems). Alternatively, insect repellents may not modify the function of the olfactory neurons directly, but as an alternative, avert other odours from stimulating olfactory neurons by dipping odour volatility at the surface of skin [17]. Since most testing for the efficacy of specific plant’s repellent effect examines only the final step in host seeking (the number of mosquito bites), these different modes of action are frequently not eminent, which can cause misperception when trying to assign a single function to an insect repellent. The specific mode of action and their major sensory receptors, and channels along with sensory appendages have been displayed (Figure 4).

**Figure 5:**
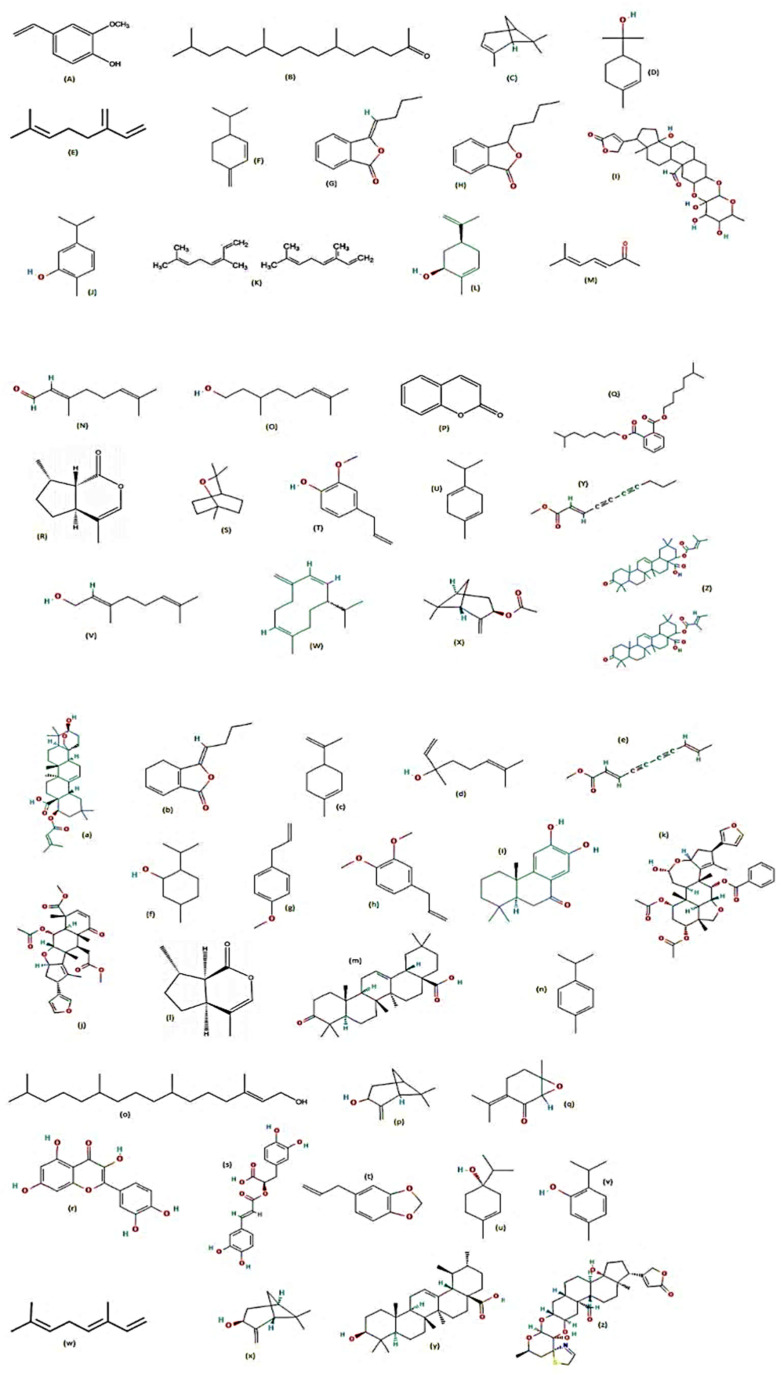
Chemical structures of major metabolites detected in plants showing repellency [74,75,77]. (**A**) 2-methoxy-4-vinyl alcohol, (**B**) 6,10,14-trimethyl-2-pentadecanone, (**C**) ⍺-pinene, (**D**) ⍺-terpineol, (**E**) β-myrcene, (**F**) β-phellandrene, (**G**) butylidene phthalide, (**H**) butylphthalide, (**I**) calotoxin, (**J**) carvacrol, (**K**) cis and trans-β-ocimene, (**L**) cis-carveol, (**M**) cis-tagetenone, (**N**) citral, (**O**) citronellol, (**P**) coumarin, (**Q**) diisooctyl phthalate, (**R**) E,Z-nepetalactone, (**S**) eucalyptol, (**T**) eugenol, (**U**) γ-terpinene, (**V**) geraniol, (**W**) germacrene D, (**X**) trans-pinocarveol acetate, (**Y**) lacnophylum ester, (**Z**) lantadene A and B, (**a**) lantanilic acid, (**b**) ligustilide, (**c**) limonene, (**d**) linalool, (**e**) matricaria ester, (**f**) menthol, (**g**) methyl carvacrol/estragole, (**h**) methyl eugenol, (**i**) nimbidiol, (**j**) nimbin, (**k**) nimbolinin A, (**l**) Z,E-nepetalactone, (**m**) oleanonic acid, (**n**) p-cymene, (**o**) phytol, (**p**) pinocarveol, (**q**) piperitenone oxide, (**r**) quercetin, (**s**) rosmarinic acid, (**t**) safrole, (**u**) terpinen-4-ol, (**v**) thymol, (**w**) trans-β-ocimene, (**x**) trans-pinocarveol, (**y**) ursolic acid, (**z**) uscharin.

The combination and blend of essential oils and extracts of some plants produced better repellency than that produced by individual extracts/oils. A few examples are: combinations of basil-peppermint oils displayed protection of 71.11% to 6.67%; nutmeg-peppermint oils showed protection of 96.67% to 3.33%; and basil-nutmeg oils showed protection of 87.77% to 0% from the 1st to the 5th hour when exposed to *Ae. aegypti* for a total of 6 h [84]. The repellent action of major plant metabolites against the dengue mosquito vector have been displayed (Figure 5 and Table 2). 

## 9. Plants Tested for Adulticidal Activity against *Aedes aegypti*

The evaluation of the adulticidal activity of the plant extracts and essential oils was carried out through the determination of the percentage of mortality of adult mosquitoes, lethal concentrations and lethal times when exposed to these extracts, oils and metabolites for a predetermined period.

**Figure 6:**
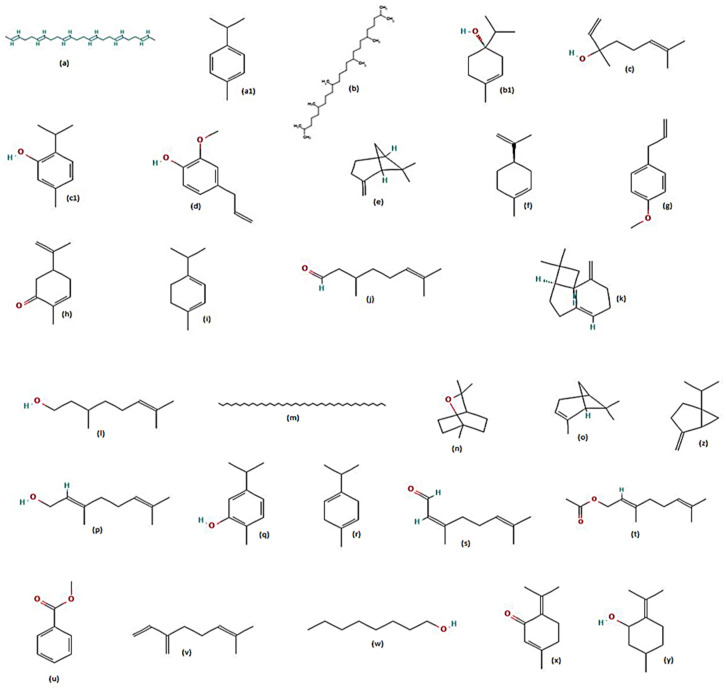
Chemical structures of major metabolites detected in adulticidal plants [73,75,77,78]. (**a**) 2,6,10,14,18,22-tetracosane hexane, (**a1**) p-cymene, (**b**) 2,6,10,15,19,23-hexamethyl tetracosane, (**b1**) terpinen-4-ol, (**c**) linalool, (**c1**) thymol, (**d**) eugenol, (**e**) *β*-pinene, (**f**) d-limonene, (**g**) estragole, (**h**) carvone, (**i**) *α*-terpinene, (**j**) citronellal, (**k**) β-caryophyllene, (**l**) citronellol, (**m**) tetracontane, (**n**) 1,8-cineole, (**o**) *α*-pinene, (**p**) geraniol, (**q**) carvacrol, (**r**) *γ*-terpinene, (**s**) neral, (**t**) geranyl acetate, (**u**) methyl benzoate, (**v**) myrcene, (**w**) octanol, (**x**) piperitenone, (**y**) pulegol, (**z**) sabinene.

The crude extracts of *Anamirta cocculus* (Menispermaceae) endosperm [67] displayed potent adulticidal activities. The PE extract produced LC_50_, LC_90_ and LC_99_ values of 140.16, 178.28 and 214.71 mg/L, respectively. While the same plant’s aqueous extract produced an LC_50_ value of 141.93 mg/L against *Ae. aegypti* after 24 h exposure [67]. The methanolic extracts of the leaves and seeds of the plant *Pithecellobium dulce* (Fabaceae) exhibited good adulticidal activities against *Ae. aegypti* when exposed for 24 h [87]. The LC_50_ and LC_90_ values for the leaves were 218.64 and 257.99 mg/L, and for the seeds, they were 426.05 and 507.73 mg/L, respectively [87]. The adulticidal action of major plant metabolites against the dengue mosquito vector have been displayed (Figure 6 and Table 3). 

## 10. Plants Tested for Pupicidal Activity against *Aedes aegypti*

The pupicidal activity of the plant extracts/essential oils was deduced depending on the mortality rates of the *Ae. aegypti* pupae when exposed to these extracts for a specific time period, following which lethal concentration and lethal time values were calculated. The essential oils extracted from four plants, namely *Alpinia galanga* (Zingiberaceae), *Anethum graveolens* (Apiaceae), *Foeniculum vulgare* (Apiaceae) and *Pimpinella anisum* (Apiaceae), exhibited good toxicity when exposed to *Ae. aegypti* pupae for a period of 72 h. At 5% concentrations, the oils of *An. graveolens* and *F. vulgare* produced 100 and 94% mortalities with LT_50_ values of 10.3 and 14.6 h, respectively [70]. At 10% concentration, *An. graveolens* displayed the highest pupicidal activity with 100% mortality and LT_50_ and LC_50_ values of 6.7 h and 2.9%, respectively [70]. The essential oils from *F. vulgare, P. anisum* and *Al. galanga* produced 99.7%, 3.5%; 98.3%, 3.84%; and 92%, 6.3% mortalities and LC_50_ values against the pupae of *Ae. aegypti*, respectively [70].

**Figure 7:**
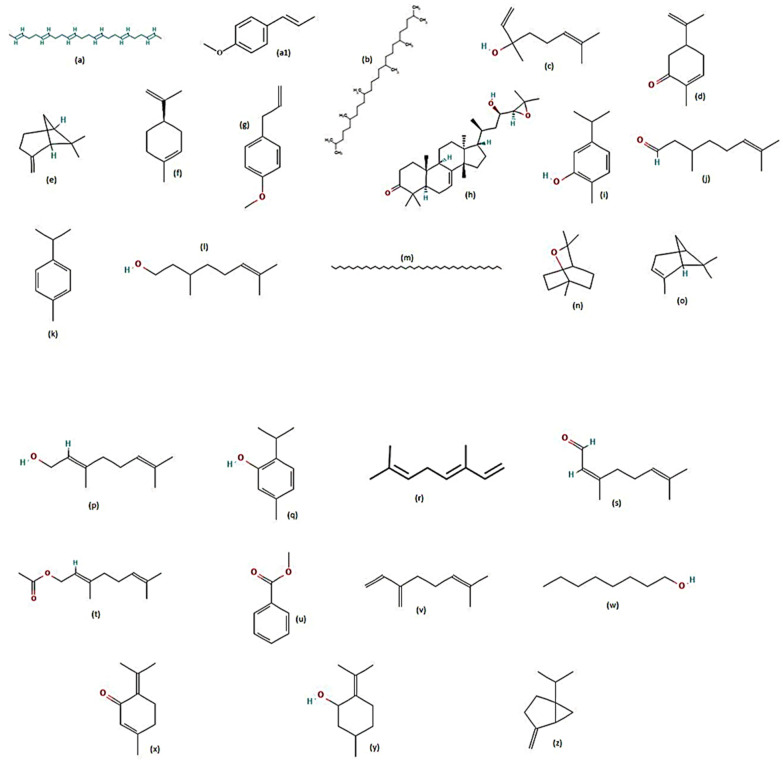
Chemical structures of major metabolites detected in pupicidal plants [73,75,77]. (**a**) 2,6,10,14,18,22-tetracosane hexane, (**a1**) trans-anethole, (**b**) 2,6,10,15,19,23-hexamethyl tetracosane, (**c**) linalool, (**d**) carvone, (**e**) β-pinene, (**f**) d-limonene, (**g**) estragole, (h) niloticin, (**i**) carvacrol, (**j**) citronellal, (**k**) p-cymene, (**l**) citronellol, (**m**) tetracontane, (**n**) 1,8-cineole, (**o**) α-pinene, (**p**) geraniol, (**q**) thymol, (**r**) trans-β-ocimene, (**s**) neral, (**t**) geranyl acetate, (**u**) methyl benzoate, (**v**) myrcene, (**w**) octanol, (**x**) piperitenone, (**y**) pulegol, (**z**) sabinene.

The crude leaf extracts of *Tribulus terrestris* (Zygophyllaceae) were pupicidal in nature [69]. This plant’s acetone extract, at concentrations of 400, 200, 100 and 50 ppm produced 57.1%, 30.8%, 23.8% and 7.7% pupal mortalities, respectively [69]. The petroleum ether extract of *Tribulus terrestris* also produced significant pupal mortalities of 100%, 100%, 82.3% and 54.5% at concentrations of 200, 100, 50 and 25 ppm, respectively [69]. The pupicidal action of major plant metabolites against the dengue mosquito vector have been displayed (Figure 7 and Table 4). 

## 11. Plants Tested for Ovicidal and Oviposition Deterrent Activities against *Aedes aegypti*

The ovicidal activity was calculated by exposing the eggs of *Ae. aegypti* to the plant extracts for a certain time period, followed by counting the number of hatched/unhatched eggs (mortality) in comparison to the number of eggs laid. In the case of oviposition deterrence, the extracts were exposed to the adult *Ae. aegypti* and % repellency was measured based on the number of eggs laid by the mosquitoes.

The hexane leaf extract of *Limonia acidissima* (Rutaceae) displayed a formidable ovicidal activity of 60% at 500 ppm against the eggs of *Ae. aegypti*, while at the same concentration, the hexane extract of *Aegle marmelos* (Rutaceae) was moderately ovicidal with an activity of 48.8% [57]. The ethyl acetate extract of *Chromolaena odorata* (Asteraceae) also produced 13.6% activity against the mosquito eggs at an identical concentration [57]. For all tested concentrations, *L. acidissima* hexane extract showed 100% oviposition deterrent activity [57]. At 500 ppm, *A. marmelos* hexane extract produced 71.79% deterrence, while the ethyl acetate extract of *Sphaeranthus amaranthoides* (Asteraceae) produced 20.48% deterrence at the same concentration [57].

**Figure 8:**
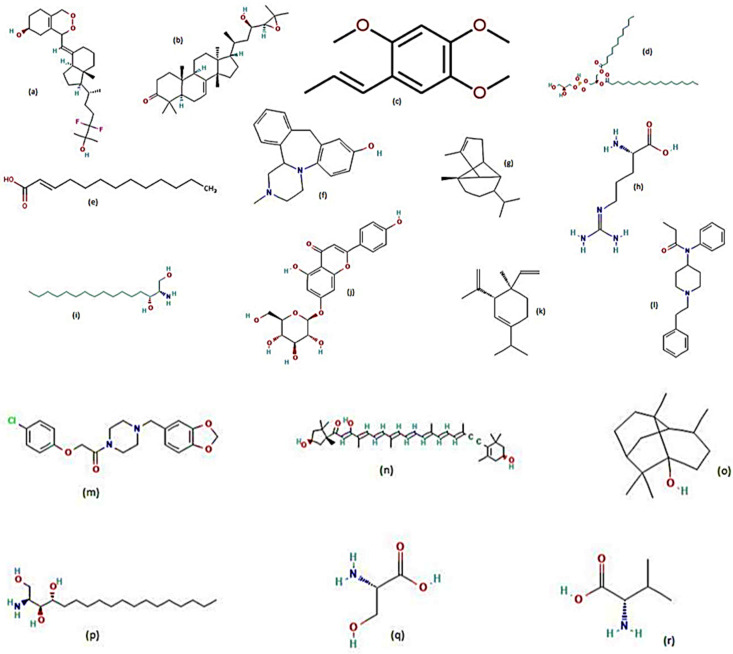
Chemical structures of major metabolites detected in plants displaying ovicidal and oviposition deterrence activities [75,76,77]. (**a**) (6RS)-6,19-epidioxy-24,24 difluoro-25-hydroxy-6,19-dihydroxyvitamin D3 (6RS)- 6,19-epidioxy-24,24-dif, (**b**) niloticin, (**c**) Z-azarone, (**d**) 1-dodecanoyl-2-octadecanoyl-glycero-3-phospho-(1′-sn-glycerol), (**e**) 3-n-decyl-acrylic acid, (**f**) 8-hydroxy mianserin, (**g**) α-copaene, (**h**) arginine, (**i**) C16 sphinganine, (**j**) cosmosiin, (**k**) δ-elemene, (**l**) fentanyl, (**m**) fipexide, (**n**) mytiloxanthin, (**o**) patchouli alcohol, (**p**) phytosphingosine, (**q**) serine, (**r**) valine.

The methanolic extract (500 mg/L) of *Rubia cordifolia* (Rubiaceae) roots produced high ovicidal activity of 70–40% against the eggs of *Ae. aegypti* [63]. The second highest ovicidal activity was shown by the hexane extract of *Scilla peruvina* (Asparagaceae) root with 43.2%, followed by hexane extract of *R. cordifolia* with 25.6% [63]. The ovicidal and oviposition deterrent action of the major plant metabolites against the dengue mosquito vector have been displayed (Figure 8 & Table 5). 

## 12. Conclusions and Future Development

This work presents detailed information on the various kinds of plant species that have been explored over the years for their mosquitocidal potential against different life cycle forms of *Ae. aegypti.*

- About 40 plant families were involved in these studies against *Ae. aegypti* and they include Schisandraceae, Rutaceae, Lamiaceae, Annonaceae, Fabaceae, Poaceae, Myrtaceae, Asteraceae, Cupressaceae, Lauraceae, Euphorbiaceae, Cucurbitaceae, Malvaceae, Linaceae, Brassicaceae, Myristicaceae, Canellaceae, Rubiaceae, Amaranthaceae, Zingiberaceae, Apiaceae, Verbenaceae, Apocynaceae, Bignoniaceae, Acanthaceae, Lythraceae, Arecaceae, Amaryllidaceae, Melastomataceae, Rhizophoraceae, Meliaceae, Asparagaceae, Celastraceae, Papaveraceae, Menispermaceae, Acoraceae, Convolvulaceae, Pinaceae, Zygophyllaceae, Anacardiaceae, Ulmaceae and Polygonaceae. All these plant families enriched with essential oils and their bio-active compounds either for its fragrance or other benefits, and the repellents that have been found to repel the dengue vector for a maximum period of 60–180 min.
- Among these, plants belonging to the Rutaceae, Lamiaceae, Asteraceae, Apiaceae, Myrtaceae, Poaceae and Fabaceae families have been frequently researched in recent times for their larvicidal, adulticidal, pupicidal and ovicidal activities against *Ae. aegypti* and other mosquito vectors.
- When compared to individual plant compounds/extracts, a blend or their combination was found to be more effective against the mosquitoes and increased the protection time [90,91,92,93,94,95,96,97].

The selection of insect repellent plants could be tailor-made, specific with the safety warnings and information about biting mosquitoes for travellers and the public and about the incidence of illness. The use of advance technologies, including effective nano-based formulation strategies with increasing repellent time, to enhance the performance of natural repellents may transform the repellent commercial market and make herbal derivatives a more feasible option for use in enduring repellents. Through the proper optimisation of repellent products by endorsing the compound binding ability to mosquitoes’ odorant receptors on the antennae and dropping their volatility, a rational and consumable pattern exists for the expansion of new bio-rational repellent formulations that could be installed as spatial repellents in effective vector control strategies across nations. Moreover, improving the technologies and cash cropping strategies of repellent herbs meet the expense of a dynamic source of revenue for agriculturalists and producers and elevate the country’s economy. Furthermore, in countries where their primary revenue depends on tourism, the practice and development of novel herbal-based repellents would increase the desire and security of travellers. As an endnote, faster work needs to be done to discover new and safe repellents for personal protection from mosquitoes. Hence, more extensive research needs to be carried out in this regard to determine the type of plant, the type of extract, the type of metabolite, the concentration and the right combinations at which they can be infused for more pronounced activity. 

## Figures and Tables

**Table 1:** Plants and metabolites that displayed significant larvicidal potency against *Ae. aegypti*.

Species/Plant Family	Part of Plant	Extract	Metabolites Detected	LC_50_	LC_90_	Time of Exposure to Larvae (hours)	Reference
*Illicium verum* (Schisandraceae)	Fruit	Essential oil	Trans-anethole	2.4 to 3.4 %	-	6	[45]
*Zanthoxylum limonella* (Rutaceae)	Fruit	Essential oil	Limonene	2.5 to 2.7%	-	6	[45]
*Origanum vulgare* (Lamiaceae)	Aerial parts	Essential oil	EO containing terpinen-4-ol, carvacrol, thymol	37.5 μg/mL	-	48	[46]
*Thymus vulgaris* (Lamiaceae)	Aerial parts	Essential oil	EO containing thymol, p-cymene, γ-terpinene	38.9 μg/mL	-	48	[46]
*Annona mucosa* (Annonaceae)	Seed	Ethanolic Extract	Rolliniatstatin 1, rollinicin	Rolliniastatin 1 = 0.43 μg/mL Rollinicin = 0.78 μg/mL	-	24	[47]
*Cymbopogon citratus* (Poaceae)	Leaves	Essential oil	EO containing citral, geranial, geraniol, β-myrcene	120.6 ppm	-	24	[48]
*Cymbopogon winteratus* (Poaceae)	Leaves	Essential oil	EO containing citronellal, citronellol, geraniol, elemol	38.8 ppm	-	24	[48]
*Eucalyptus citriodora* (Myrtaceae)	Leaves	Essential oil	EO containing citronellal, citronellol, isopulegol	104.4 ppm	-	24	[48]
*Eucalyptus camaldulensis* (Myrtaceae)	Leaves	Essential oil	EO containing 1,8-cineole, α-pinene, citronellyl acetate	33.7 ppm	-	24	[48]
*Achillea bieberstenii* (Asteraceae)	Aerial parts	Essential oil	EO containing α-terpinene, p-cymene	EO = 23.6 μL/L α-terpinene = 70.1 μL/L	-	24	[49]
*Juniperus procera* (Cupressaceae)	Aerial parts	Essential oil	EO containing eugenol, β-caryophyllene	EO = 12.2 μL/L Eugenol = 38.3 μL/L	-	24	[49]
*Annona glabra* (Annonaceae)	Leaves	Extract	Silver nanoparticles (An-AgNPs)	5.945 mg/L for 24 h 3.5485 mg/L for 48 h	-	24, 48	[50]
*Brassica napus* (Brassicaceae)	Leaves	Essential oil	Lipids and fatty acid methyl esters	Fatty acid methyl esters = 342.8 ppm	-	24	[51]
*Pavetta tomentosa* (Rubiaceae)	Fresh Leaves	Extract	2,6,10,14,18,22-tetracosane hexane; 2,6,10,15,19,23-hexamethyltetracosane	Crude extract = 5.96 μg/mL	Crude extract = 7.49 μg/mL	24	[52]
*Tarenna asiatica* (Rubiaceae)	Fresh Leaves	Extract	Tetracontane	Crude extract = 1.28 μg/mL	Crude extract = 1.99 μg/ml	24	[52]
*Ambrosia arborescens* (Asteraceae)	Leaves	Extract	Silver nanoparticles	0.28 ppm	0.43 ppm	24	[53]
*Pinus sylvestris* (Pinaceae)	Needles	Essential oil	3-cyclohexane-1-methanol, alpha, alpha.4-trimethyl	EO = 100.39mg/L	-	24	[54]
*Syzygium aromaticum* (Myrtaceae)	Buds	Essential oil	Eugenol, eugenyl acetate	EO = 92.56 mg/L	-	24	[54]
*Mentha villosa* (Lamiaceae)	Leaves	Essential oil	EO containing rotundifolone	EO = 45 ppm Rotundifolone = 62.5 ppm	-	24	[55]
*Carum carvi* (Apiaceae)	Voucher specimen	Essential oil	Carvone, limonene, γ-terpenene	EO = 54.62 ppm	-	24	[56]
*Apium graveolens* (Apiaceae)	Voucher specimen	Essential oil	D-limonene, phthalides	EO = 42.07 ppm	-	24	[56]
*Foeniculum vulgare* (Apiaceae)	Voucher specimen	Essential oil	Trans-anethole, D-limonene, estragole	EO = 49.32 ppm	-	24	[56]
*Zanthoxylum limonella* (Rutaceae)	Voucher specimen	Essential oil	D-limonene, terpinen-4-ol, sabinene	EO = 24.61 ppm	-	24	[56]
*Curcuma zedoaria* (Zingiberaceae)	Voucher specimen	Essential oil	1,8-cineole, p-cymene, α-phellandrene	EO = 31.87 ppm	-	24	[56]
*Limonia acidissima* (Rutaceae)	Leaves	Extract	Niloticin	0.44 ppm	1.17 ppm	24	[57]
*Mentha arvensis* (Lamiaceae)	Fresh Leaves	Essential oil	(Corn mint oil) containing menthol, methyl acetate, menthone and limonene	78.1 ppm	125.7 ppm	24	[58]

**Table 2:** Plants and their metabolites that displayed significant repellency against *Ae. aegypti*.

Plant Species/Family	Part of the Plant	Metabolites Detected	% Repellency	% Protection/Duration of Protection	Complete Protection Time (CPT)	Time of Exposure to theMosquito	Ref.
*Origanum* *vulgare* (Lamiaceae)	Aerial parts	Terpinen-4-ol, carvacrol, thymol	8.9% to 37.8% (Essential oil)	-	-	24 h	[46]
*Thymus* *vulgaris* (Lamiaceae)	Aerial parts	Thymol, p-cymene, γ-terpenes	4.4% to 68.9% (Essential oil)	-	-	24 h	[46]
*Ateleia* *glazioviana* (Fabaceae)	Leaves	Flavonoids	84.5% (Dichloroethane extract)	-	-	24 h	[59]
*Ocimum* *basilicum* (Lamiaceae)	Leaves	Eucalyptol, linalool, eugenol	70.5% (Alcoholic spray derived from essential oil)	-	-	24 h	[59]
*Myristica* *fragrans* (Myristicaceae)	Nutmeg oil	α-pinene, terpinen-4-ol, safrole	-	100% protection for first 2 h, 90 to 23.32% for the next 4 h	-	6 h	[84]
*Mentha piperita* (Lamiaceae)	Peppermint oil	Menthol	-	96.67% to 27.78% for the first 4 h	-	6 h	[84]
*Ocimum* *basilicum* (Lamiaceae)	Basil oil	Methyl chavicol, geraniol, methyl eugenol	-	98.9% to 2.22% for 5 h	-	6 h	[84]
*Chenopodium ambrosioides* (Amaranthaceae)	Aerial parts	Trans-pinocarveol, cis-carveol, trans-pinocarvyl acetate	39.7% (Essential oil)	-	-	5 min	[85]
*Conyza sumatrensis* (Asteraceae)	Aerial parts	Cis-lachnophyllum ester, limonene, trans-β-ocimene	51.4% (Essential oil)	-	-	5 min	[85]
*Erigeron canadensis* (Asteraceae)	Aerial parts	Limonene, matricaria ester	80% (Essential oil)	-	-	5 min	[85]
*Eucalyptus camaldulensis* (Myrtaceae)	Fresh leaves	Pinocarveol, myrtenol, β-phellandrene	13.7% (Essential oil)	-	-	5 min	[85]
*Mentha spicata* (Lamiaceae)	Aerial parts	Piperitenone oxide, eucalyptol	100% (Essential oil)	-	-	5 min	[85]
*Parthenium hysterophorus* (Asteraceae)	Aerial parts	Germacrene-D, β-myrcene, trans-β-ocimene	63.9% (Essential oil)	-	-	5 min	[85]
*Targetes minuta* (Asteraceae)	Aerial parts	Cis-β-ocimene, cis-tagetenone, limonene	50.2% (Essential oil)	-	-	5 min	[85]
*Nepeta cataria* (Lamiaceae)	CR9, CR3 crude essential oils	*Z, E*-nepetalactone and *E, Z* nepetalactone isomers	10% CR9 crude oil showed >95% repellency for the first 2 h	-	-	24 h	[41]
*Cymbopogon citratus* (Poaceae)	Leaves	Citral, limonene, α-terpineol, citronellol	-	-	The blend of all extracts was used For 1% *w/v*-1 h 2% *w/v*-2 to 3 h 5% *w/v*-5 to 6 h	-	[40]
*Lantana Camara* (Verbenaceae)	Leaves	Oleanonic acid, lantadene A&B, lantanilic acid					
*Calotropis gigantea* (Apocynaceae)	Leaves	Acetate, citrates, chloride					
*Ocimum sanctum* (Lamiaceae)	Leaves, flowers, branches	Eugenol, ursolic acid, rosmarinic acid					
*Azadirachta indica* (Meliaceae)	Leaves	Nimbolinin, nimbin, quercetin, nimbidiol					
*Calotropis Procera* (Apocynaceae)	Leaves	Uscharin, calotoxin, calotropeol acetate					
*Angelica sinensis* (Apiaceae / Umbelliferae)	Rhizome and root	3-N-butylphthalide, butylidenephthalide, di-iso-octyl phthalate, ligustilide	-	-	2.5 h (EO) 2.5 h (Ethanolic extract) 7.5 h (Hexane extract) 1.75 h (Acetone extract) 0.5 h (Methanolic extract)	Every 3 min in a 30 min interval	[86]
*Mentha arvensis* (Lamiaceae)	Fresh leaves	Essential oil (corn mint oil) containing menthol, methyl acetate, menthone, and limonene	-	-	25% EO–45 min 90 min (50% EO) 165 min (100% EO)	-	[58]

Note: The essential oil *Hierochloe odorata* (Poaceae) was extracted from its leaves by and was found to contain metabolites such as phytol, coumarin, 2-methoxy-4-vinyl alcohol and 6,10,14-trimethyl-2-pentadecanone. The repellency of this plant’s EO, hexane and ethanol crude extracts were determined using PNB, and their PNB values were found to be 0.84, 0.48 and 0.44, respectively.

**Table 3:** Plants and metabolites that displayed significant adulticidal activity against *Ae. aegypti*.

Species/Plant Family	Part of Plant	Metabolites Detected	LC_50_	LC_90_	% Mortality	Time of Exposure to the Mosquito	Reference
*Origanum vulgare* (Lamiaceae)	Aerial parts	EO containing terpinen-4-ol, carvacrol, thymol	14.3 μg/mL	-	-	90 min	[46]
*Thymus vulgaris* (Lamiaceae)	Aerial parts	EO containing thymol, p-cymene, γ-terpinene	11.7 μg/mL	-	-	90 min	[46]
*Achillea bieberstenii* (Asteraceae)	Aerial parts	EO containing α-terpinene, p-cymene	30.2 μL/L (EO) 66.8 μL/L (α-terpinene) 54.1 μL/L (p-cymene)	-	-	24 h	[49]
*Juniperus procera* (Cupressaceae)	Aerial parts	EO containing eugenol, β-caryophyllene	10.1 μL/L (EO) 18.3 μL/L (Eugenol) 46.4 μL/L (β-caryophyllene)	-	-	24 h	[49]
*Pavetta tomentosa* (Rubiaceae)	Fresh leaves	2,6,10,14,18,22-tetracosane hexane; 2,6,10,15,19,23-hexamethyltetracosane	32.105 μg/mL (Crude extract)	41.001 μg/mL (Crude extract)	-	60 min	[52]
*Tarenna asiatica* (Rubiaceae)	Fresh leaves	Tetracontane	9.012 μg/mL (Crude extract)	11.854 μg/mL (Crude extract)	-	60 min	[52]
Lippia alba (Verbenaceae)	Dry whole plant (voucher specimen)	Limonene, carvone, piperitenone	-	-	24% (at 390 ppm of EO after 24 h)	24 h	[88]
*Lippia origanoides* (Verbenaceae)	Dry whole plant (voucher specimen)	Carvacrol, p-cymene, thymol	-	-	68% (at 300 ppm of EO within 2 min)	24 h	[88]
*Eucalyptus citriodora* (Myrtaceae)	Dry whole plant (voucher specimen)	Citronellol, pulegol, citronellal	-	-	13% (at 390 ppm of EO after 24 h)	24 h	[88]
*Cymbopogon flexuosus* (Poaceae)	Dry whole plant (voucher specimen)	Neral, geraniol, geranyl acetate	-	-	>92% (at 1000 ppm of EO after 60 min)	24 h	[88]
*Citrus sinensis* (Rutaceae)	Dry whole plant (voucher specimen)	Limonene, myrcene, n-octanol	-	-	76% (at 390 ppm of EO after 24 h)	24 h	[88]
*Cananga odorata* (Annonaceae)	Dry whole plant (voucher specimen)	Methyl benzoate, linalool	-	-	>92% (at 1000 ppm of EO after 60 min)	24 h	[88]
*Swinglea glutinosa* (Rutaceae)	Dry whole plant (voucher specimen)	α-pinene, β-pinene, sabinene,1,8- cineole	-	-	>92% (at 1000 ppm of EO after 120 min)	24 h	[88]
*Tagetes lucida* (Asteraceae)	Dry whole plant (voucher specimen)	Myrcene, estragole, trans-β-ocimene	-	-	>92% (at 1000 ppm of EO after 24 h)	24 h	[88]

**Table 4:** Plants and metabolites that displayed significant pupicidal potency against *Aedes aegypti*.

Plant Species/Family	Part of Plant	Metabolites Detected	LC_50_	LC_90_	% Mortality	LT_50_	Time of Exposure to the Mosquito	Reference
*Illicium verum* (Schisandraceae)	Fruit	Trans-anethole	-	-	86.4 to 100% (5% trans-anethole)	6.9 to 28.8 h (5% trans-anethole) 1.5 to 5.2 h (2.5% EO + 2.5% trans-anethole)	72 h	[45]
*Zanthoxylum limonella* (Rutaceae)	Fruit	Limonene	-	-	89.6 to 94.4% (5% d-limonene))	23.8 to 28.5 h (5% d-limonene) 7.9 to 15.3 h (2.5% EO + 2.5% d-limonene)	72 h	[45]
*Pavetta tomentosa* (Rubiaceae)	Fresh leaves	2,6,10,14,18,22-tetracosane hexane; 2,6,10,15,19,23 -hexamethyltetracosane	For 24 h, Acetone extract = 1.361 μg/mL; Hexane extract = 2.044 μg/mL; Chloroform extract = 2.512 μg/mL For 48 h, Acetone extract = 3.273 μg/mL; Hexane extract = 1.682 μg/mL; Chloroform extract = 2.298 μg/mL	-	-	-	24 and 48 h	[52]
*Tarenna asiatica* (Rubiaceae)	Fresh leaves	Tetracontane	For 24 h, Acetone extract = 1.682 μg/mL; Hexane extract = 1.990 g/mL; Chloroform extract = 2.429 μg/mL For 48 h, Acetone extract = 4.555 μg/mL; Hexane extract = 3.008 μg/mL; Chloroform extract = 3.975 μg/mL	-	-	-	24 and 48 h	[52]
*Lippia alba* (Verbenaceae)	Dry whole plant (voucher specimen)	Limonene, carvone, piperitenone	-	-	24% and 29% after 24 and 48 h (at 390 ppm of EO)	-	24 and 48 h	[88]
*Lippia origanoides* (Verbenaceae)	Dry whole plant (voucher specimen)	Carvacrol, p-cymene, thymol	-	-	67% and 73% after 24 and 48 h (at 250 ppm of EO)	-	24 and 48 h	[88]
*Eucalyptus citriodora* (Myrtaceae)	Dry whole plant (voucher specimen)	Citronellol, pulegol, citronellal	-	-	13% and 47% after 24 and 48 h (at 390 ppm of EO)	-	24 and 48 h	[88]
*Cymbopogon flexuosus* (Poaceae)	Dry whole plant (voucher specimen)	Neral, geraniol, geranyl acetate	-	-	13% and 47% after 24 and 48 h (at 390 ppm of EO)	-	24 and 48 h	[88]
*Citrus sinensis* (Rutaceae)	Dry whole plant (voucher specimen)	Limonene, myrcene, n-octanol	-	-	27% and 42% after 24 and 48 h (at 390 ppm of EO)	-	24 and 48 h	[88]
*Cananga odorata* (Annonaceae)	Dry whole plant (voucher specimen)	Methyl benzoate, linalool	-	-	27% and 56% after 24 and 48 h (at 390 ppm of EO)	-	24 and 48 h	[88]
*Swinglea glutinosa* (Rutaceae)	Dry whole plant (voucher specimen)	α-pinene, β-pinene, sabinene,1,8- cineole	-	-	38% and 73% after 24 and 48 h (at 390 ppm of EO)	-	24 and 48 h	[88]
*Tagetes lucida* (Asteraceae)	Dry whole plant (voucher specimen)	Myrcene, estragole, trans-β-ocimene	-	-	56% and 67% after 24 and 48 h (at 390 ppm of EO)	-	24 and 48 h	[88]
*Limonia acidissima* (Rutaceae)	Leaves	Niloticin	0.62 ppm (Niloticin) 4.19 ppm (Hexane extract)	1.45 ppm (Niloticin) 8.10 ppm (Hexane Extract)	-	-	24 h	[57]

**Table 5:** Plants and metabolites that displayed significant ovicidal and oviposition deterrent activities against *Aedes aegypti*.

Plant Species/Family	Part of the Plant	Metabolite Detected	Ovicidal Activity (% Mortality)	Oviposition Deterrent Activity (Number of Eggs Laid)	Time of Exposure to the Eggs/Adult *Aedes aegypti*	Ref.
*Limonia acidissima* (Rutaceae)	Leaves	Niloticin	83.2% (at 2 ppm of niloticin)	-	120 h	[57]
*Cyanthocline purpurea* (Asteraceae)	Leaves	3-n-decyl acrylic acid, C16 sphinganine, mytiloxanthin	>70% (ethanolic extract at 0.2 mg/mL)	-	48 h	[89]
*Blumea lacera* (Asteraceae)	Leaves	Phytosphingosine, cosmosiin, valine, serine, arginine	≈75% (ethanolic extract at 0.1 mg/mL)	-	48 h	[89]
*Neanotis lancifolia* (Rubiaceae)	Leaves	Fentanyl, 8-hydroxy mianserin, 1-dodecanoyl-2-octadecanoyl-glycero-3-phospho-(1′-sn-glycerol)	90% (ethanolic extract at 0.1 mg/mL)	-	48 h	[89]
*Neanotis montholonii* (Rubiaceae)	Leaves	(6RS)-6,19-epidioxy-24,24 difluoro-25-hydroxy-6,19-dihydrovitamin D3/(6RS)-6,19-epidioxy-24,24-dif, 1-dodecanoyl-2-octadecanoyl-glycero-3-phospho-(1′-sn-glycerol), fipexide	≈90% (ethanolic extract at 0.1 mg/mL)	-	48 h	[89]
*Piper marginatum* (Piperaceae)	Leaves and stem	D-elemene, α-Copaene, patchouli alcohol, (Z)-asarone	-	<50% eggs laid (at 50 and 100 ppm of leaf and stem extracts) <40% eggs laid (essential oil)	14 h	[89]

## Data Availability

Not applicable.

## Supplementary Materials

The following supporting information can be downloaded at: https://www.mdpi.com/article/10.3390/molecules28052386/s1, Figure S1: Different extraction processes for plant repellent substances.

## Abbreviations

DSS	Dengue Shock Syndrome
DHF	Dengue Haemorrhagic Fever
DEN-2	Dengue Virus Type-2
STI	Sterile Insect Technique
DEET	N, N-diethyl-meta-toluamide
Lao PDR	Lao People’s Democratic Republic
CDIAL	Cinnamodial
CHIKV	Chikungunya Virus
CML	Cinnamolide
CMOS	Cinnamosmolide
CPCD	Capsicodendrin
DDD	Dichlorodiphenyldichloroethane
DDT	Dichlorodiphenyltrichloroethane
DENV EO	Dengue Fever Virus Essential Oil
GOI	Government of India
ICMR	Indian Council of Medical Research
LC	Lethal Concentration
NCBI	National Center for Biotechnology Information
NIV	National Institute of Virology
PMD	Para-menthane-3,8-diol
POLYG	Polygodial
RNA	Ribonucleic Acid
UGAN	Ugandensolide
USD	United States Dollars
VRDL	Virus Research & Diagnostic Labs
WARB	Warburganal
WHO	World Health Organization
YFV	Yellow Fever Virus
ZIKV	Zika Virus
ZVD	Zika Virus Disease
