# Supplementary material for: Bioactive Molecules Derived from Plants in Managing Dengue Vector Aedes aegypti (Linn.)
Source: Molecules. 2023 Mar 5;28(5):2386. doi: 10.3390/molecules28052386 (PMC10005433; doi:10.3390/molecules28052386)
Supplement: Supplementary file 1 [file molecules-28-02386-s001.zip › molecules-2215383-supplementary.pdf]

Supplementary Material

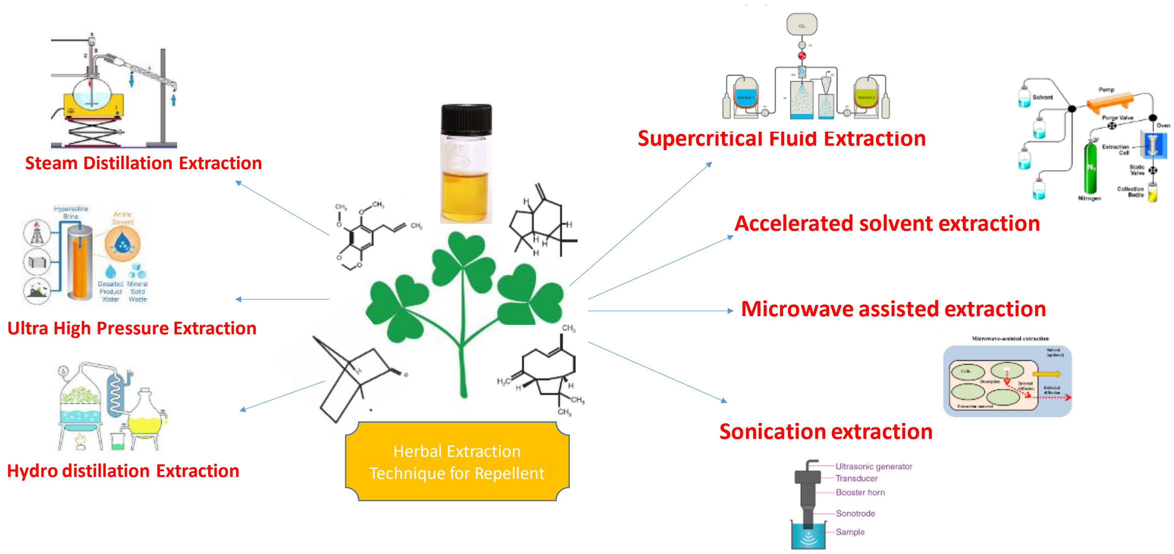

Figure S1. Different extraction process for plant repellent substances.
